# Cell-Tissue Interaction: The Biomimetic Approach to Design Tissue Engineered Biomaterials

**DOI:** 10.3390/bioengineering10101122

**Published:** 2023-09-25

**Authors:** Paola Nitti, Athira Narayanan, Rebecca Pellegrino, Stefania Villani, Marta Madaghiele, Christian Demitri

**Affiliations:** Department of Engineering for Innovation, University of Salento, 73100 Lecce, Italy; athira.narayanan@unisalento.it (A.N.); rebecca.pellegrino@unisalento.it (R.P.); stefania.villani@unisalento.it (S.V.); marta.madaghiele@unisalento.it (M.M.); christian.demitri@unisalento.it (C.D.)

**Keywords:** extracellular matrix, cell-tissue interactions, tissue engineering, regenerative medicine, scaffolds

## Abstract

The advancement achieved in Tissue Engineering is based on a careful and in-depth study of cell–tissue interactions. The choice of a specific biomaterial in Tissue Engineering is fundamental, as it represents an interface for adherent cells in the creation of a microenvironment suitable for cell growth and differentiation. The knowledge of the biochemical and biophysical properties of the extracellular matrix is a useful tool for the optimization of polymeric scaffolds. This review aims to analyse the chemical, physical, and biological parameters on which are possible to act in Tissue Engineering for the optimization of polymeric scaffolds and the most recent progress presented in this field, including the novelty in the modification of the scaffolds’ bulk and surface from a chemical and physical point of view to improve cell–biomaterial interaction. Moreover, we underline how understanding the impact of scaffolds on cell fate is of paramount importance for the successful advancement of Tissue Engineering. Finally, we conclude by reporting the future perspectives in this field in continuous development.

## 1. Introduction

Degeneration or loss of organ and/or tissue function due to injury, disease, or ageing has a tremendous impact on quality of life and poses a large social and economic cost. Annually, billions of U.S. dollars are spent to perform surgical procedures to restore damaged tissues and organs. Therefore, in the last fifty years, new strategies have emerged to overcome these problems like Tissue Engineering (TE) and Regenerative Medicine (RM) [1]. These strategies promote the regeneration of damaged or diseased tissues and organs using the synergistic action of biomaterial-based scaffolds, growth factors, and cells [2]. It is essential to understand how tissues naturally recover when employing a TE approach, as well as the actors, mechanisms, and signals involved in processes that occur spontaneously in tissues [3]. This knowledge allows the design of scaffolds that best mimic the characteristics of the native tissue and therefore promotes new tissue formation or regeneration.

In tissues and organs, the extracellular matrix (ECM) is an essential extracellular element that surrounds cells, characterised by its sophisticated nanoarchitecture. It is a highly hydrated structure composed of cell-secreted proteins (e.g., collagen, fibronectin, elastin, etc.), macromolecules (e.g., polysaccharides, hyaluronan, glycosaminoglycans—GAGs—and proteoglycans—PGs), and specialised soluble factors (e.g., ions, growth factor, cytokines, and hormones) [4].

ECM provides structural and mechanical support in which cells can adhere and operate but, above all, it offers a broad spectrum of biophysical (e.g., stiffness, topography, viscoelasticity, etc.) and biochemical (e.g., receptor targeting ligands, pH, soluble signalling factors, etc.) cues that regulate vital cellular functions such as survival, adhesion, migration, proliferation, self-renewal, differentiation, morphogenesis, and gene expression [5]. In particular, cell expression of protein-receptors, like integrins, on their plasmatic membrane allows binding to the ECM and initiates a cascade of many cellular and tissue processes that influence regeneration. Therefore, understanding how cells interact with the ECM is crucial to obtain a biomaterial-based scaffold that allows cells to colonise and interact with the biomaterial as they naturally do with ECM, therefore leading to regeneration processes. TE scaffolds should evoke the native ECM, providing mechanical support and direct tissue development. To achieve this goal, the strategy is to design and manufacture scaffolds with specific characteristics and nanoarchitecture like native ECM, resulting in increased biological interactions between cells and biomaterial, thereby supporting cell infiltration, adhesion, differentiation, and oxygen and nutrient transport [6,7,8]. The two main functionalization approaches are bulk and surface functionalization. The tailoring of the biomaterial surface is of particular interest to improve interactions between cells/tissue and scaffolds. The surface is the scaffold’s part that is in direct contact with the human body so it is decisive for the performance and host acceptance of the scaffold [3]. Specific properties of biomaterials, such as hydrophilicity, free energy, roughness, softness, chemical composition, and morphology, influence cell–scaffold interactions and the success of the healing process. In recent years, many studies have focused on surface modifications for the development of biocompatible and bioactive biomaterial scaffolds without altering the bulk material properties [9], like the immobilisation of functional groups and active biomolecules, or permeability and mechanical properties modification.

Unfortunately, today it is still difficult to obtain all the desired scaffold’s characteristics due to fabrication techniques, which present many limitations, and materials that do not present anchorage molecules to most mammalian cells and often lack biocompatibility and bio-functionality [3,10]. In addition, there is still a lack of knowledge about ECM and its mechanisms, which limits the possibility of designing a proper microenvironment for cells. For this reason, it is necessary to increase the research in this field.

Firstly, this review attempts to define the organisation of native ECM and how cells answer to the matrix interaction (via a Unit Cell Process), and then define the cell–biomaterial interactions focusing on material physical and chemical properties and their modification to improve cell–biomaterial interactions and, therefore, the regenerative processes.

## 2. ECM: A Key Player for TE

The ECM composition can vary among tissue types, resulting in several phenotypes that confer tissue specificity in physical and mechanical properties. In addition, ECM composition can be modified in response to intrinsic and extrinsic factors, giving rise to a dynamic and responsive niche for cells and tissues [11].

### 2.1. ECM Structure

The structural organisation of ECM includes two layers: the pericellular matrix and the interstitial matrix. The pericellular matrix is a well-organised network in close contact with the overlying cells by establishing cross-junctions with integrins, Discoidin Domain Receptors (DDRs), and peptidoglycans [12]. A classic example of a pericellular matrix is represented by the Basement Membrane (BM) [13], an adhesive microenvironment that provides biochemical and physical support to resident cells. Its main molecular components are collagen type IV, laminins, nidogen 1 and 2, and PGs such as perlecan, agrin, collagen type XV, and collagen type XVIII [12,14]. Epithelial cells (ECs) can adhere to BM thanks to specific structures called hemidesmosomes, formed by the interactions of cell surface integrins and intermediate filaments with laminins [12,15]. The interstitial matrix is generally more porous and less dense than the overlying BM. It is mainly composed of collagens, elastin, and fibronectin, creating a final 3D amorphous gel [13].

### 2.2. ECM Components

ECM composition can vary among tissue types and can be influenced by development stage, age, and pathology [5]. Its components are classified into (1) fibrillar, structural, and adhesive proteins (collagen, elastin, laminin, fibronectin, vitronectin); (2) amorphous matrix macromolecules (PGs, GAGs, hyaluronan); and (3) specialised soluble factors (growth factors, cytokines, hormones) [5].

Collagens are the most abundant components in the ECM. They are synthesised mainly by fibroblasts, representing up to 30% of the total proteins in humans, creating a 3D network of fibres in both pericellular and interstitial matrices [12]. Twenty-eight different collagen types are responsible for creating a 3D network of fibres in the pericellular and interstitial matrix [16]. Collagens are classified into seven types: types I, II, III, V, XI, XXVI, and XXVII are the most abundant among tissues and they maintain a fibrillar organisation, whereas types IV, VIII, and X form networks and supramolecular structures by interacting with other ECM components [16]. Collagens are often exploited in TE to create collagen-based biomaterials to be used in sports medicine and wound healing [17]; however, the role of collagens in the ECM for physiological and pathological tissue conditions is still being studied [18]. Elastin is an adhesive component of the ECM found in specific tissue types, where it is responsible for adequate tissue elasticity [16] and tissue stretching recovery [13]. It is constituted by tropoelastin monomers that interact by self-assembling to finally obtain mature elastic fibres, and then they cross-link with an outer layer of fibrillin microfibrils, creating an elastic fibre [13]. Laminins are a class of heterotrimeric cross-shaped glycoproteins localised in the BM [12,13]. Besides being crucial during embryonic development, laminins play a role in cellular processes like differentiation, migration, and adhesion, ensuring the survival of tissues [12]. Fibronectin (FN) is localised in the BM, and it is responsible for cellular adhesion and wound healing processes [13,19]. It can exist in two different forms: the soluble plasmatic form is in the blood to be delivered to the site of injury, and the cellular form is synthesised by fibroblasts [13]. Cells can assemble FN by taking soluble molecules from the blood or synthesising it autonomously. FN fibrils can interact with the actin cytoskeleton of cells through a class of surface receptors called integrins, finally forming fibrils with a thickness between 10 and 100 nm [13]. Vitronectin (VNT), also known as S-protein or serum diffusion factor, is an adhesive glycoprotein that is located between cells and the ECM, where it interacts with several ligands like integrins, plasminogen activator inhibitor-1 (PAI-1), and the urokinase plasminogen activator receptor (uPAR) [20]. VNT works as a multimeric complex (unfolded or active form) in the ECM of several tissue types [20], where it promotes ECs adhesion and tissue remodelling [21]. Dysfunction and misfolding of VNT can promote the development of neurodegenerative diseases, such as age-related macular degeneration, Alzheimer’s disease, and multiple sclerosis, showing the essential role played by the ECM [21].

GAGs are polar carbohydrates composed of repeating disaccharide units of *N*-acetylated hexosamines (*N*-acetyl-d-galactosamine or *N*-acetyl-d-glucosamine) and d-/l-hexuronic acid (d-glucuronic acid or l-iduronic acid) [12]. GAGs are divided into four groups based on their carbohydrate residues: hyaluronic acid (HA), chondroitin sulfate (CS) and dermatan sulfate (DS), heparan sulfate (HS), and keratan sulfate (KS) [5]. HA is a linear GAG made by repeating disaccharide units of d-glucuronic acid and *N*-acetyl-d-glucosamine found in the ECM with or without a protein core. HA is a major constituent of the pericellular matrix where it can adsorb substantial amounts of water molecules by affecting tissue elasticity [12]. In mammals, there are three HA synthase (HAS) isoforms responsible for HA synthesis, whereas for hyaluronidases degrade HA, the combination of these two enzymatic activities could affect HA size and molecular weight [12]. GAGs interact with core proteins to finally form PGs, which are localised not only in the ECM, but also in intracellular compartments and at the cell surface, influencing some cellular processes like proliferation, migration, differentiation, apoptosis, and adhesion. The PGs interactions with growth factors, cytokines, and cell surface receptors, either via their core proteins or through their GAGs, are essential for the formation of an ECM 3D scaffold [12]. PGs can be classified into four families: intracellular, cell surface, pericellular, and extracellular membrane. Extracellular PGs are the most abundant and they are divided into two subgroups: hyalectans, which include aggrecan, versican, neurocan, and brevican; small leucine-rich PGs, like decorin, are the largest family of PGs containing eighteen members divided into five classes ubiquitously expressed in most ECMs [12]. Pericellular PGs, like perlecan and agrin, are often associated with cells through integrin cell receptors. Syndecans and glypicans are the two main subfamilies of cell surface PGs that link ECM components with the cellular surface [12]. Serglycin is the only characterised intracellular PG, and it is present not only in hematopoietic cells, where it manages the storage and the packaging of bioactive molecules, but also in ECs and smooth muscle cells, chondrocytes, fibroblasts, and tumour cells, modulating their aggressiveness [12].

Growth factors, cytokines, and hormones localised in the ECM can modulate cellular functions through biochemical interactions. The specific growth factors present in the ECM can be different among tissue types and for physiological and pathological conditions. However, one of the most common growth factors is represented by the Transforming Growth Factor-β (TGF-β), a family of homodimeric or heterodimeric secreted cytokines. These proteins are synthesised in a native form that is cleaved during the secretory pathway, leading to the formation of a mature dimeric ligand bounded via a single disulfide bond [22,23]. TGF-β is stored in the matrix together with the latent TGF-β binding protein (LTBP) in an inactive form. Once it is activated, it can regulate ECM remodelling and it can promote a fibroblast to myofibroblast transition, which is essential to induce the fibrotic process [24]. Some ECM macromolecules can directly bind soluble factors, for example, decorin binds TGF-β, modulating its bioavailability, but also vascular endothelial growth factor (VEGF), insulin-like growth factor I (IGF-I), and platelet-derived growth factor (PDGF) [25]. Parallelly, FN shows some binding sites for epidermal growth factor (EGF), VEGF, and hepatocyte growth factor (HGF), modulating the migration and metabolism of ECs [26].

### 2.3. Cellular Adhesion to the ECM

The interactions between ECM and adherent cells are mediated by a family of transmembrane proteins called integrins. In addition to ensuring cell anchorage to the matrix and making contact by binding FN, laminin, collagen, and cellular receptors, they also provide cell–cell interactions [27]. Integrins are heterodimers of α and β subunits. Humans express eighteen α and eight β subunits that, when combined, can generate twenty-four different integrin heterodimers with overlapping but non-redundant functions [27]. The integrins’ activation requires some structural rearrangements to modulate the affinity for ligands, like the activation of the proteins talin and kindlin, and the negative regulators ICAP-1α and filamin [28]. The balance between activated and inactivated integrins controls cell adhesion and polarity. In certain classes of ECs, a complex called hemidesmosome, which includes α6β4 integrins, serves as a linkage between intermediate filaments and adherent cells. In addition, a second major bond between ECs and the underlying BM is represented by integrins-containing focal adhesions that, unlike hemidesmosomes, connect the actin cytoskeleton to the BM through indirect integrin–actin connections [27,29]. Focal adhesions also mediate some transduction pathways like cytoplasmic alkalinization, can increase intracellular calcium, activate tyrosine kinases, protein tyrosine phosphatases, and lipid kinases, and modulate gene expression [6].

Although integrins are the most studied, ECM possesses other families of macromolecule receptors including the DDR family for collagens, CD44 and Receptor for HA-Mediated Motility (RHAMM) for HA, and HS PG-like syndecans for various other ECM molecules [26]. The DDR family includes DDR1 and DDR2, whose ligands are collagen I–III, while DDR1 can only recognise collagen IV [30]. The main CD44 ligand is HA, but also osteopontin [31,32]. Syndecans’ ligands are collagen I, III, and V, FN, and laminin, and can also interact with other integrins and cell adhesion receptors [33].

## 3. Exploring ECM Biophysical and Biochemical Properties for Enhanced TE

The evaluation of ECM biophysical and biochemical properties is essential for TE in the optimization of 3D matrices for in vitro and in vivo applications. As mentioned before, ECM is a dynamic environment, whose properties greatly influence the cellular fate specifically based on the tissue type.

### 3.1. ECM Biophysical Properties

ECM components strongly modulate the tissue response to mechanical forces. Collagens are responsible for ECM strength and stiffness, and they reach a strength of ~0.12 GPa and an elastic modulus of ~1.2 GPa in mammalian tendons [34,35]. Collagen fibres exhibit great energy storage but only moderate (13%) stretchability due to their hierarchical organisation [34,36]. Additionally, tissue strength could be impacted by a fibre’s thickness. Generally, collagen type III is thinner and more flexible than type I and their ratio varies among tissue types, affecting tissue mechanical properties [34,37]. The ECM elasticity is related to its stiffness, defined as the stress (force per unit area) needed to induce a given strain (deformation) [38]. It has been demonstrated that an increase in deposition and cross-linking of collagen and HA molecules could affect ECM stiffness, and the mechanical conduction to resident cells could modulate their biological behaviour [39]. In more detail, HA interacts with the HA receptor CD44, while collagen components bind integrin receptors, modulating the ECM stiffness [40] and, consequently, inducing some biological pathways like glucose, lipid, and amino acid metabolisms [39], and cancer metastasis [41,42]. Durotaxis is the process by which some cell types, like fibroblasts, cancer cells, mesenchymal stem cells, and ECs, could sense the substrate stiffness, preferring to migrate from soft to stiff matrices [43]. Also, cells can exploit focal adhesion sites to sense the ECM rigidity by applying local forces, modulating cell adhesion and migration [44].

However, ECM in biological tissues not only behaves as an elastic material, but it has some features in common with viscous liquids. The term viscoelasticity refers to the ECM characteristic of having a solid-like elastic response followed by a time-dependent liquid-like viscous behaviour [38]. The type and strength of the bonds that crosslink the ECM could affect its viscoelasticity. Weak bonds among ECM macromolecules facilitate stress relaxation through fibre displacement and energy dissipation, while covalent bonds impede ECM plastic deformation, balancing ECM stiffness and viscoelasticity. Together with the strength of the chemical bonds and the molecular weight of the polymeric components, could affect ECM viscoelasticity: low molecular weight molecules interrupt the ECM network, promoting stress relaxation and energy dissipation. This aspect has been exploited in the design of polymer-based matrices with alginate [45] and HA [46] to affect cell proliferation, migration, and gene expression [47]. Indeed, it has been demonstrated that ECM with fast stress relaxation promotes filopodia-based migration and 3D adhesion [48]. Together with ECM stiffness and viscoelasticity, makes its topography a fundamental parameter to be taken into consideration for biomedical engineering. Native ECM possesses nanoscale physical topographies [49] and porosity [50]. These intrinsic features could be transferred to engineered scaffolds by creating micro- and nano topography to control cell adhesion, migration, differentiation, and morphology [51].

### 3.2. ECM Biochemical Properties

The ECM structure and components could affect the dynamic relationship between cells and the environment during the adhesion process. It has been demonstrated that a lower ECM density reduces adhesions’ formation because the adhesion mechanism is affected by the level of intracellular contractility [52]. Together, with the composition, allows ECM rheological properties to modulate the adhesion dynamics.

ECM shows electric properties that differ among tissue types, depending on the fluid content of the matrix: blood and brain conduct electric current relatively well, while lungs, skin, fat, and bone are poor conductors. Due to technical restrictions on the use of electrodes for biological investigations, there is limited information regarding the conductivity and electric characteristics of biological tissues [44]. Data on the muscle–skeletal system are the most abundant, but due to the anisotropy of these tissues, it is necessary to distinguish between transverse and longitudinal directions, complicating the measurements. In the case of tumour tissues, they generally show different electrical conductivity and permittivity than physiological tissues, and this aspect could be exploited for the tumour diagnosis. The skin is one of the most resistive tissues, with an impedance that is dominated by the stratum corneum [44]. Especially for cardiac TE, the matrix’s conductivity is an essential aspect to be considered. A biomimetic scaffold has to mimic the conductivity of the heart muscle, and some strategies have been optimised to reproduce the electric properties of the human tissue by the incorporation of conductive or carbon-based particles or by using conductive polymers [53].

As a dynamic microenvironment, ECM is subject to remodelling processes induced by variations in density, composition, stiffness, and degradation. ECM degradation is a common process that occurs with the intent to balance qualitatively and quantitatively the composition of the ECM. However, these dynamics are often associated with the development of some pathological states like cancer [54], chronic liver disease [55], and metabolic diseases [56]. The main responsible components of ECM remodelling by degradation are the matrix metalloproteinases (MMPs), a family of twenty-three zinc-dependent enzymes that show increased activity in pathological conditions [57]. Based on their distribution and molecular affinity, MMPs are divided into membrane-type MMP (MT-MMP), collagenases (MMP-1, MMP-8, MMP-13, and MMP-18), gelatinases (MMP-2 and MMP-9), stromelysins (MMP-3, MMP-10, MMP-11), matrilysins (MMP-7 and MMP-26). MT-MMPs and collagenases degrade triple-helical collagen molecules, while gelatinases recognise the basal lamina fibres, causing cell death; stromelysins and matrilysins remodel ECM by degrading segments and components of the matrix [58]. MMPs have three common domains: pro-peptide, catalytic, and hemopexin-like C terminal domain. The latter is responsible for substrate specificity, and it is linked to the catalytic domain by a flexible hinge region called “linked region”. The pro-peptide domain interacts with the catalytic zinc in the active site, inhibiting the substrate binding and, therefore, keeping the enzyme in the inactive form. The proteolytic cleavage can cut the pro-peptide domain, activating the MMPs [59]. The ECM remodelling exercised by MMPs, as well as guaranteeing the correct matrix homeostasis, is, therefore, able to modulate cell fate in terms of adhesion, migration, and differentiation.

## 4. Modulation of Cell Fate by Cell–Biomaterial Interactions

Thanks to the recent progress registered in the fields of materials science and TE, it is possible to modulate the physicochemical and biological properties to generate a bio-based scaffold for various applications, from antibacterial surfaces [60] and engineered bacteria [61] to tissue regeneration [62,63,64,65,66] and wound healing [17,67,68]. Particularly, cell–biomaterial interactions are crucial to determine the cellular fate in terms of adhesion [69,70], proliferation [71], differentiation [72,73], morphology [74,75], migration [76,77], and for the ECM-mimetic scaffold fabrication [78,79].

### 4.1. Biomaterials

According to the International Union of Societies for Biomaterials Science and Engineering (IUSBSE), a biomaterial is defined as “a material designed to take on a form that can guide the course of any therapeutic or diagnostic operation through interactions with biological systems” [80]. Since biomaterials can directly interact with cells and tissues, the foundation for constructing materials with regenerative ability rests on understanding the cellular mechanism involved in the interaction between cells and material. The characteristics of biomaterials and their behaviour towards the surroundings are crucial for the performance and acceptance of cells because they stimulate greater cell adherence, which leads to additional multi-cellular responses. The physicochemical and biological characteristics of the material control the qualitative and quantitative adsorption of proteins, especially ECM proteins, which influence cell attachment mediated by adhesion receptors such as integrins. The dynamic connection of cells with the ECM, assisted by integrins and controlled by material surface properties, initiates signal transduction, which leads to biological responses of cells such as proliferation, differentiation, or cytokine release [3,81].

The biophysical and biochemical signalling pathways present in the cellular responses are influenced by the physical characteristics of biomaterial [82]. In addition, the chemical and biological properties of the material are also equally important to have a better interaction with cells. The chemical characteristics of the material influence many cellular functions: adhesion, proliferation, and differentiation, as well as determining the adsorption, composition, and conformation of the ECM [83]. The synthesis of a biomaterial with specific characteristics for TE systems has exhibited a higher affinity towards cells compared to native material. The functionalization techniques that are used for the design and manufacture of scaffolds with desired characteristics mainly involve bulk and surface functionalization [9]. In biology, reactions usually occur at the interface, not in solution, hence, the surface properties of both the cell and the biomaterial play a key role in the cell–biomaterial interactions. The increased accessibility for reactions offered at the surface promotes complex reactions, molecular recognition, and specific molecular orientation, and also enhances reaction turnover rates [84]. Thus, surface properties of biomaterials are considered a promising area for improving cell–biomaterial interactions without any change in their bulk properties, which can be accomplished using a variety of physicochemical and biological processes [85,86]. These methods depend on the use and type of material. Biological surface modification techniques are extensively employed due to their strong effects on cell interactions, whereas the chemical and physical surface modifications obtained further consideration in recent years [9]. Surface chemistry deals with the material interfaces’ chemical characteristics and other surface modifications [87]. The recent techniques, mainly used for the surface modification of a biomaterial and their effect on the cell response, are presented in Table 1. Similar to surface properties, bulk properties are crucial parameters that specify the physicochemical qualities of the material over the lifespan of the designed structures [9]. Hence, the modulation of in vivo and in vitro cell responses, such as adhesion, cell cycle progression, survival, and expression of differentiated phenotypes, as well as regulation of cell–host interactions and biological integration, is influenced by the surface as well as bulk properties of biomaterials [88,89].

### 4.2. Unit Cell Process

**Figure 1:**
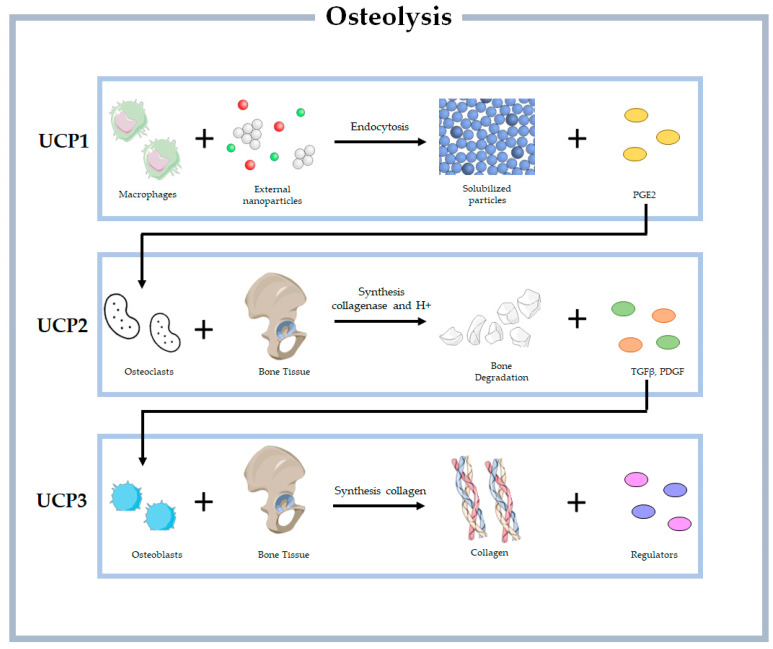
Schematic representation of UCPs involved in osteolysis, in which UCP2 is predominant.

In TE and RM, the use of biomaterials plays an important role in manipulating cell functions and providing a micro-environment that allows the seeded cells to adhere and differentiate into the desired tissue, facilitating cellular processes that are indispensable for tissue regeneration [119,120]. To simplify cell–matrix interactions, biomaterials in the form of scaffolds, fillers, and prostheses can be considered as stimuli to activate cells and induce them to perform certain functions such as proliferation, migration, ECM assembly, differentiation, endocytosis, exocytosis, and apoptosis [121]. The first five functions are important to stimulate tissue regeneration. On the other hand, cells adhere to the substrate and can perceive it as a regulator. This kind of identification takes place thanks to integrins, which recognise the external environment, transferring specific signals to the internal one and vice versa [122]. Cells can also exert forces on the scaffold, remodelling it. Significant challenges still exist in understanding the complexity of interactions between biomaterials and cellular behaviour. For simplicity, it would be better to study a cell function as if it comprises several distinct processes to better know them and try to regulate them through the use of external factors. This approach is based on the definition of the Unit Cell Process (UCP), that is, each cell function is activated by an external regulator, which could be physiological or provided by the external biomaterial used. In this way, a specific cell–matrix interaction can be described by employing UCP, defining the cell type of interest and possible regulators involved. For example, a soluble regulator such as TGF-β, in combination with mechanical stimuli provided by the scaffold, could activate fibroblasts in connective tissue to assemble a new ECM. In this process, new cytokines would be released and those in turn will activate other processes. More complex cellular responses can be described by the combination of two or more UCPs. This is what happens, for example, when unstable insoluble prostheses are implanted in vivo, causing the release of external particles in the surrounding tissue. In this case, an excessive presence of these particles would be the starting point for the activation of a series of cell processes. Briefly, macrophages would be activated to destroy these particles by endocytosis; in their action, they would release signals, such as prostaglandin E2 (PGE2), which activate osteoclasts for bone degradation through the synthesis of collagenase and the release of H^+^ ions. In this process, although growth factors such as TGF-β and PDGF would activate osteoblasts for the synthesis of new collagen, there is still an imbalance that results in osteolysis (Figure 1). In this way, by the definition of UCPs, it is possible to describe what happens when cells come in contact with an external biomaterial and therefore interfere in this process in a specific way.

**Figure 2:**
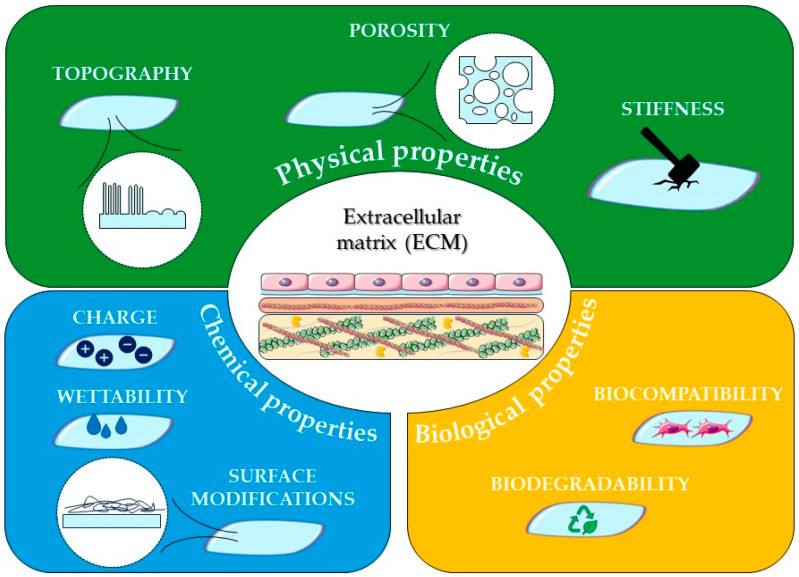
Schematic representation of physical, chemical, and biological properties of polymeric scaffolds that can be modulated to optimise the interactions with the ECM.

In TE and RM, the aim of all efforts is the regeneration of new tissue and the integration of the biomaterial (used in different forms) in vivo. For this reason, knowing the target tissue and the processes involved in its regeneration, it would be possible to use a specific biomaterial in the most appropriate way to regulate those processes by interacting with cells and modulating their functions (Figure 2).

## 5. Physical Properties of Biomaterials

### 5.1. Orientation and Porosity

Among new approaches in the field of TE, the use of scaffolds is achieving more success. These 3D structures allow cellular adhesion and growth, the formation of the new tissue, and its final form. Therefore, scaffolds are necessary to guide and facilitate cellular processes that are indispensable for tissue regeneration. They serve as a framework to support cell migration into the defect from surrounding tissues and as a delivery vehicle for exogenous cells, growth factors, and genes. They also preserve the defect site and tissue volume, avoiding distortions and the collapse of the surrounding tissue, and they act as a barrier against bacterial infiltrations that are dangerous for tissue regeneration. Isolated and expanded cells adhere to a temporary scaffold in all three dimensions, proliferate, and secrete their ECMs, replacing the biodegrading scaffold. The study of Soleas et al. demonstrated that the scaffold’s physical properties directly interfere with cell differentiation. Progenitor cells cultured in polydimethylsiloxane (PDMS) tubes could self-organise into tube structures, suggesting that the geometry of the scaffold interferes with cell morphology, depending on the diameter of the tube, and determining their fate status due to constraints imposed [72]. Designing and creating scaffolds provide significant challenges since their physical characteristics are one of the most important variables affecting interactions between cells and biomaterials.

To facilitate the formation of the desired new tissue, scaffolds might mimic its specific characteristics. Therefore, it is necessary to understand the complexity of the target tissue and try to reproduce it through the scaffold [123]. For example, several kinds of tissues present a highly oriented morphology. Numerous studies have shown that the use of scaffolds with oriented structures can influence cell shape and distribution, and ECM arrangement. Therefore, having oriented scaffolds results in aligned cells with a higher aspect ratio of nuclei and a well-oriented ECM arrangement [124]. In this way, this specific arrangement of scaffolds’ structure can be used in these cases. For the regeneration of muscle fibres, it is evident that a key factor is the alignment of muscle cells in a specific direction. To verify that the presence of such orientation in the scaffold could effectively induce a direct effect on cell distribution; Hoon Yang et al. modified a 3D printed polycaprolactone (PCL)-based scaffold by a stretching process to obtain an aligned pattern. By comparing stretched and unstretched scaffolds and their interaction with cells, they noted that modified scaffolds showed more elongated cells aligned along the pattern and an increase in their proliferation and differentiation with the formation of a greater number of myotubes [125]. In blood vessels, ECs in the intima layer show a specific distribution along the longitudinal axis. For this reason, Niu et al. fabricated random and aligned electrospun fibres tubular scaffolds with mechanical properties that matched those of native vessels, and they compared the effect of the fibres’ orientation on cells. Although cell proliferation was good on each scaffold, cell morphologies changed from polygonal in random conformation to spindle-like in oriented ones. In the latter case, they were also parallel to fibres and more like cells in native tissue [126]. The study of Li et al. aimed to mimic the multi-layered cell-specific orientation of blood vessels using a dual oriented/bilayered small-diameter tubular scaffold fabricated by electrospinning, using a mixture of PCL, poly (D, L-lactide-co-glycolide) (PLGA), and gelatin. The orientation of nanofibres exerted contact guidance for cell distribution, with slenderer paving-stone-like morphologies of both smooth muscle cells (SMCs) and ECs and F-actin spread along the cell-oriented direction, in contrast to random scaffolds in which cells did not have a preferential orientation and F–actin was disordered [127].

Having an anisotropic structure could also help in nerve injury repair. Ghaderinejad et al. successfully fabricated an injectable anisotropic alginate hydrogel for nerve TE by adding short PCL nanofibres containing superparamagnetic iron oxide nanoparticles, which allow fibres to align directly in situ in the presence of an external magnetic field. In aligned hydrogels, it was possible to achieve higher proliferation of human olfactory ecto-mesenchymal stem cells (OE-MSCs) and higher levels of marker genes for neural differentiation [128].

To promote cellular growth, the optimal cell distribution in the structure, and the neovascularization of the new tissue, scaffolds should also have a high porosity, i.e., a high specific surface area or area/volume ratio [129,130]. The presence of pores plays a crucial role in the fabrication of effective scaffolds used in TE and RM because they permit the transport of gases and nutrients, as well as the removal of waste molecules due to cellular metabolism. As a result, it is necessary to have an appropriate mean pore size: if pores are too small, cells cannot penetrate the scaffold, and the diffusion of nutrients and waste is limited, which would lead to necrotic regions within the construct. In contrast, if pores are too large there is a decrease in surface area, limiting cell adhesion and compromising structural integrity and mechanical strength. Therefore, it is important to maintain a balance between the optimal pore size for cell migration and the specific surface area for cell attachment. Pores should also be interconnected to allow an optimal spatial cell distribution throughout the scaffold to facilitate homogeneous tissue formation. For example, Jia et al. fabricated porous magnesium (Mg) scaffolds for bone TE by modulating the pore size and distribution. Although mechanical strength decreases with the increase of pore size and interconnectivity, the degradation rate was not affected and cell migration, as well as cell viability and proliferation, were enhanced [131]. The porosity of a scaffold can be tailored based on the specific TE application and desired outcomes. For example, longitudinally aligned pores were obtained in the study of Basurto et al. to mimic the anisotropic architecture of muscle fibres. These 3D collagen scaffolds were fabricated using directional lyophilization to obtain a specific direction of pores and conductive polypyrrole (PPy) nanoparticles to enable electrically excitable myotube assembly and maturation. Confocal images of both the longitudinal and transverse scaffold planes confirmed that the scaffold’s microstructure would influence cell alignment. In fact, in the transverse plane, where pores were isotropic and rounded, cells were randomly distributed. In contrast, in the longitudinal plane, myoblasts showed anisotropic cytoskeletal alignment. The oriented porosity in scaffolds could effectively facilitate cytoskeletal organisation along a specific direction, increasing metabolic activity and similarity to healthy skeletal muscle [132].

### 5.2. Topography

It has been proved that cells can recognise micro- and nano-scale changes in the environment, thus a scaffold’s topography can influence cellular responses, in particular, their morphology and distribution [133]. Topography refers to the physical surface features of the scaffold’s material, which can be manipulated from the point of view of texture, roughness, pattern, and geometry. The modification of such characteristics represents an active area of research intending to improve cell migration, proliferation, and differentiation as well as guide specific cellular responses [124]. For instance, it was demonstrated that nano- and micro-patterned surfaces can influence cell shape. When adhered to a patterned surface with a particular geometry, the shape of cells adopts the same one [134]. This can also influence cellular activities: some studies have demonstrated that a specific surface scaffold’s topography can induce stem cell differentiation into desired cell lineages [134]. Stem cells are commonly used in TE and RM thanks to their proliferative capacity and the possibility to differentiate [135]. Unfortunately, achieving the proper differentiation of stem cells is not simple since, in vivo, they are subjected to numerous biochemical and biophysical signals that are difficult to replicate with the scaffold. Therefore, having the opportunity to regulate their behaviour holds great promise in this field. For example, aligned features on the surface will promote muscle–skeletal differentiation, obtaining cells with elongated morphology [136]. Yang et al. fabricated a polystyrene (PS) scaffold with microgroove patterns by using a combination of near-field electrospinning (NFE) and template lithography. In the first step, they deposited poly (ethylene oxide) (PEO) fibres by NFE, then they poured PS solution on the PEO fibres template and dried them. After the removal of the PEO template in water, they obtained a patterned PS substrate. Cell viability and proliferation assays demonstrated that cells were elongated in the pattern direction, so microgrooves can effectively guide cell growth and orientation through pattern alignment [137].

In addition to having a specific orientation on the surface of scaffolds, it is possible to modify their roughness to obtain better cellular growth. Increasing surface roughness results in an increase of specific surface area, thus providing greater sites for cell adhesion on the scaffold and an increase in their proliferation. Although there is a large number of studies about the effect of topographical features on cellular activities, the findings are often controversial due to the use of different cell types, which can act differently [138].

In bone TE, enhancing the adhesion of cells on scaffolds is a key factor in starting cell differentiation and the formation of new tissue. In this context, the surface roughness of constructs could be helpful. Zhang et al. fabricated porous bioceramic β-tricalcium phosphate (β-TCP) scaffolds for bone TE, using an in situ growth crystal process to manipulate surface topography and to study its effect on stem cell behaviour. Modified scaffolds with micro- and nano-crystals on the surface, thus obtaining a greater surface roughness, showed better cell adhesion and morphology with a large amount of fusiform cytoskeleton, and enhanced phosphorylation of Extracellular signal-Regulated Kinases (ERK), c-Jun N-terminal Kinases (JNK), and Signal Transducer And Activator Of Transcription 3 (STAT3), finally promoting osteogenesis [139]. The importance of roughness was also confirmed by the study of Shams et al. They fabricated nanofibrous polyether sulfone scaffolds and modified their surface by using fluorapatite nanoparticles. In this way, they obtained an increase in hydrophilicity and roughness, which resulted in better proliferation and differentiation of human bone marrow mesenchymal stem cells (hBMMSCs) [140].

### 5.3. Stiffness

Scaffold stiffness, related to its mechanical properties and elasticity, is an important characteristic of TE and RM. It indicates the ability of a scaffold material to resist deformation under an applied force or stress, thus indicating the rigidity of the substrate [141]. Scaffold stiffness plays a significant role in influencing cell behaviour, tissue development, and overall tissue-engineered construct functionality. The mechanical properties of a scaffold can have a specific impact on cellular processes, including cell adhesion, proliferation, migration, and differentiation. Cells in the first stage can sense substrate features, such as stiffness, in a process called mechano-sensation. Then, they respond to the mechanical cues provided by the scaffold’s stiffness, converting mechanical forces into biochemical signals that regulate cellular behaviour in a process called mechano-transduction [141,142]. It is now well known that a scaffold’s stiffness can influence stem cell differentiation, but also cell migration, enhancing the penetration of tissue cells into the scaffold itself. In addition, it can influence cell morphology and cytoskeletal organisation. Numerous studies have demonstrated that if the substrate is softer, cells will obtain a rounded morphology, in contrast to stiffer substrates where cells will spread more easily [142].

Different tissues in the body have varying levels of stiffness or elasticity. For example, soft tissues, like the brain or the adipose tissue, exhibit low stiffness, while hard tissues such as bone have high stiffness. It is important to note that the optimal scaffold’s stiffness depends on the specific tissue being targeted and the intended application. In some cases, mimicking the native tissue’s stiffness can be beneficial for cell behaviour and tissue integration. For example, some studies have shown that if the stiffness of the scaffold matches that of the target tissue, it is possible to guide stem cell differentiation in the specific lineage of interest [134]. In other instances, adjusting the scaffold stiffness to provide mechanical cues that promote desired cellular responses, such as osteogenesis in bone TE, may be necessary.

The stiffness of a scaffold can be modulated by selecting suitable scaffold materials and adjusting their composition, structure, and fabrication methods. For instance, using different polymer formulations, crosslinking densities, or incorporating reinforcement materials like fibres or nanoparticles can influence the scaffold’s stiffness. Overall, scaffold stiffness is a critical design parameter that must be carefully considered and tailored to create an appropriate microenvironment for cells, facilitating tissue development and promoting successful tissue regeneration in TE applications.

Lee et al. modulated the compressive stiffness of collagen-GAG scaffolds by using four different cross-linking methods: dehydrothermal treatment (DHT), ultraviolet irradiation (UV), glutaraldehyde treatment (GTA), and 1-ethyl-3-(3-dimethyl aminopropyl) carbodiimide (EDC). In each case, chondrocyte proliferation and the synthesis of a new matrix were evaluated, although, for each scaffold, the DNA content increased over time; higher stiffness of the substrate was due to chemical cross-linking of EDC, which resulted in higher cell proliferation. In addition, protein and GAG synthesis were higher. Therefore, it emerged that modulating the stiffness of the scaffold resulted in more stability over time. It could be seen that scaffolds cross-linked with the use of EDC could contrast the action of the intracellular contractile proteins, presenting the lowest diameter reduction [143].

On the other hand, Zhang et al. utilised 3D bioprinting to fabricate scaffolds using a combination of alginate, gelatin, and human MSCs as low-cost bio-ink. They modulated the stiffness of such scaffolds to know their influence on osteogenic differentiation and tissue formation over time. The analysis showed that softer scaffolds had higher DNA content, enhanced alkaline phosphatase (ALP) activity and stimulated osteogenic differentiation, obtaining, over time, more mineralized tissue and higher osteoblast and early osteocyte-related gene expression [144].

## 6. Chemical Properties of Biomaterials

### 6.1. Surface Reactive Functional Groups

The common chemical functional groups used for altering the surface chemistry of biomaterials are -CH_3_, -NH_2_, -COOH, OH, -CO-, -CO_3_^2−^ [82,145,146]. The immobilisation of biomolecules or biomaterials onto the surface of constructs is usually conducted through chemical grafting of functional moieties like aminolysis, hydrolysis, acetylation, silanization, fluorination, and sulfonate incorporation [147]. Chemical reactions, like reduction and oxidation, can also be used to modify the functional groups already present in the biomaterial. The introduction of cross-linking agents, like EDC/N-Hydroxysuccinimide (NHS), maleimide, avidin-biotin, and click chemistry reactions, boosted the surface functionalization techniques by improving the efficiency of chemical reactions between the biomaterials and biomolecules [148,149,150], as well as providing specific-controlled conjugation, respectively [151,152].

Self-assembled monolayers (SAM) of ω-functionalised alkanethiols on gold were used to study the human MSCs differentiation with different surface chemistry enabled by four functional groups -CH_3_, -COOH, -NH_2_, and -OH [153]. The results showed that the amino group functionalised SAMs promoted osteogenic and adipogenic differentiation relative to all other functionalised surfaces. The experiments on silane functionalised surfaces were used to characterise the behaviour and the differentiation of bone marrow-derived MSCs, which demonstrated that -NH_2_ and -SH functionalised surfaces supported and maintained osteogenesis, while -OH and -COOH modified surfaces stimulated chondrogenesis, and -CH_3_ modified surfaces preserved MSC phenotypes [146]. A study on biomaterial interactions with human embryonic stem cells (hESCs) was performed with 576 different combinations of 25 different acrylate, diacrylate, dimethacrylate, and triacrylate monomers with a radical initiator onto a layer of poly (hydroxyethyl methacrylate) (pHEMA) [154]. The results showed that cell attachment and spreading differ from the monomers: certain monomers inhibited growth on hESCs, whereas almost all the monomers prefer to be grown on C2C12 cells (an embryonic muscle cell line). A well-defined surface with different functional groups (-CH_3_, -NH_2_, -COOH, and -OH) was created using alkanethiol-based SAM techniques for the investigation effect of surface chemistry on human dental pulp stem cells (hDPSCs), and it was observed that -NH_2_ functionalised surfaces showed a highly branched osteocyte-like morphology with improved cell focal adhesion, proliferation abilities, and enhanced osteo/odontogenesis differentiation potential [155]. They also found that the surface functionalised with other groups maintained the MSCs-like phenotype. Studies on rabbit bone marrow stromal cells (BMSCs) cultivated on substrate functionalised with -NH_2_ group showed an enhancement in the mRNA expression and osteogenic differentiation of the BMSCs [156]. Moreover, –NH_2_- and –OH-modified substrates were well spread and homogeneous with the actin organised into stress fibres and demonstrated long microtubules and prominent focal adhesions, but the -COOH- and -CH_3_ modified substrates resulted in a more rounded phenotype. The effect of surface chemistry on neural stem cells (NSCs) demonstrated that cells behave differently towards each functionalised surface [157]. The -NH_2_ and -OH groups showed an active interaction with cells and triggered the signalling pathways of adhesion, migration, proliferation, and division. At the same time, -OH groups downregulated the cell metabolism while -NH_2_ groups induced the expression of genes associated with axon growth. CH_3_ groups displayed fewer interactions with the membrane receptors and maintained the property of NSCs. Other studies on mesoporous bioactive glass modified with an amino group (N-MBG) showed an enhancement in the osteogenic differentiation of BMSCs and upregulation of anti-inflammatory cytokines, as well as an inhibition of the formation of tartrate-resistant acid phosphatase (TRAP) positive multinuclear cells in macrophages [158]. Human foreskin fibroblasts, cultured on ultra-high molecular weight polyethene (UHMWPE) surface incorporated with amine moieties using allylamine-based plasma and UV techniques, promoted cell adhesion and proliferation [159].

Despite having excellent properties, cytotoxicity is a challenge for single-walled carbon nanotubes (SWCNTs). The studies conducted on the HepG2 cell line confirmed that the hydroxyl group functionalised short SWCNTs might be safer than the others and provide great value for the risk assessment and application of SWCNTs [160]. Studies on amino-functionalised SWCNT/PCL scaffolds produced via electrospinning have proved the progress in the adhesion, proliferation, and differentiation of rat bone marrow-derived MSC [161]. The bioactive glass scaffolds, modified with the -SH and -NH_2_ groups using the post-grafting technique, significantly stimulated the adhesion, proliferation, and differentiation of hBMMSCs [162]. The effect of surface chemistry on fibronectin adsorption force (F_ad_) was examined on SAMs [163]. SAMs were terminated with functional groups using the Au-thiol method, observing that F_ad_ on SAMs followed a chemistry dependence of -NH_2_ > -CH_3_ >> -OH. The fibronectin adsorption force and conformation can control the late osteoblast adhesion and subsequent reorganisation of adsorbed FN and fibrillogenesis of the endogenous FN.

The use of cross-linkers has been widely explored in cell–biomaterial interactions to have a better reaction between the functional groups of biomolecules and biomaterials. EDC/NHS has been commonly used for the chemical interaction between the amine group of biomolecules and the carboxylic group of biomaterial surface [164] due to its non-cytotoxicity and water solubility of byproducts [149]. PCL/poly (m-anthranilic acid) (P3ANA) electrospun nanofibres were functionalised with RGD (arginyl glycyl aspartic acid) peptide in which the -COOH groups in the aniline backbone of P3ANA obtain covalently attached with surface-activated RGD peptide using an EDC/NHS linker, reported to enhance attachment, proliferation and osteogenic activity of Saos-2 cells [165]. Covalent attachment between carboxylic groups of GAGs and amine groups of collagens in GAGs-collagen matrices obtained by the EDC/NHS method has been employed to improve the scaffold resistance to enzymatic degradation in human Wharton’s Jelly-derived ECM (WJ-ECM)-based scaffolds for skin wound healing [166]. Implantable dopamine moieties grafted HA hydrogel (HA-DOPA) scaffolds with encapsulated human adipose-derived stem cells (hASCs) in the bulk, and hESCs-corneal limbal epithelial stem cells (LESCs) on the surface were synthesised, which imparted good tissue adhesive properties, facilitated the covalent conjugation with the cell-adhesive proteins to the hydrogel surface and supported the regeneration of corneal epithelium and stroma cells [167]. Scaffolds made from 3D freeze-dried gelatin and electrospun PLGA fibres were coated with hydroxyapatite nanoparticles (HAn), followed by crosslinking through an EDC/NHS solution, and enhanced osteoblast proliferation [168]. The maleimide reactive group has been known for its selective reactivity to cysteine residues in proteins and it is widely used for the immobilisation of biomolecules on various metallic and glass surfaces [169,170]. The thiol–maleimide reaction has received increasing attention for providing good cell–biomaterial interactions involving thiol-containing biomolecule surfaces [171,172,173].

Hydrogel-based drug delivery systems made up of maleimide functionalised HA (HA-Mal) and gelatin (Gel-Mal) crosslinked with a bifunctional thiolated polyethene glycol (PEG) crosslinker were examined for regenerative applications [174]. Genipin, a green crosslinker, displays excellent biocompatibility, admirable biodegradability, and stable cross-linked attributes. It can only react with primary amine groups rather than secondary and tertiary amino groups and has been explored to produce various genipin-crosslinked biomaterials [175,176,177]. Chitosan-polyvinyl alcohol (PVA)-Genipin cross-linked films induced accelerated healing, quick fibroblast generation, and angiogenesis, affirming their suitability for wound healing applications [178].

### 6.2. Surface Charge

The biomaterial surface can possess charges either neutral, positive, or negative via the functional groups already present, or by using different mechanisms such as adsorption of ions, dissociation of surface chemical groups, and application of external electric field in aqueous solutions [3,179]. A better cell–biomaterial response through increased protein adsorption and conformation is achieved by introducing the proper surface charge on the biomaterial by the targeted molecule and the cell type [180]. N-MBG has been reported as a good platform for the MC3T3-E1 cell adhesion, proliferation, and differentiation due to the positive charge distributed by the -NH_2_ group, making it a promising material for bone TE [181]. MSCs from the bone marrow were seeded onto a PEG hydrogel surface coated with four different chemical groups using the alkane–thiol method. The results validated that free neutral surfaces (-CH_3_ and -OH) led to greater chondrogenic induction extent but less protein adsorption, cell spreading, and adhesion than charged surfaces (-NH_2_ and -COOH) [182]. Negatively charged carboxymethyl chitosan-gelatin (CMCG) composite membranes fabricated via anodic electrophoretic deposition (AED) have demonstrated their ability to transport drugs or other medical agents containing negative charges, which suggested that CMCG membranes could act as a strong candidate for surface functionalised biomaterials with negative charges [183]. The ε-poly-L-lysine (EPL) and phenylboronic acid (PBA)-modified gelatin methacrylamide hydrogels (GelMA-EPL and GelMA-EPL/B) synthesised via Michael addition reaction exhibited positive surface charges, significantly promoting adsorption of negatively charged PGs and secreted PGs in the solution and hence providing a good 3D microenvironment for cartilage repair with improved biocompatibility [184]. Furthermore, GelMA-EPL/B hydrogel enhanced the formation of many ECMs. A dynamic UV-triggered pH-responsive surface was constructed on titania nanotubes (TNTs) by loading photoacid generators, diphenyl iodonium chloride, followed by grafting 2,3-dimethyl maleic anhydride (DMMA)-modified hyperbranched poly(l-lysine) (HBPLL) onto the surface [185]. The low pH developed after the UV irradiation led to the dissociation of DMMA and, thereby, the transformation of surface chemistry from negatively charged carboxyl groups to positively charged amino groups. The TNTs–HBPLL–DMMA substrate confirmed that it could better promote the proliferation and spreading of rat bone MSCs after UV irradiation. The 3D-printed Alginin (Alg)/ε-Polylysine (ε-PL) scaffold-charged surfaces were found to be capable of facilitating the controllable immobilisation and release of CS or growth factors, thus improving the proliferation and chondrogenic differentiation of hBMSCs [186]. The cross-linking between the negatively charged -COOH group of Alg and positively charged amine group of ε-PL enhanced the mechanical stability and by adjusting the stoichiometric ratio of Alg and ε-PL, as well as the amount of additional ε-PL, and the surface charge of the scaffolds can be tuned, hence controllable degradation behaviour would be produced.

### 6.3. Surface Wettability

Surface wettability is considered a measurement of surface energy, which is used to describe the ability of water droplets to maintain contact with the solid surface [187,188]. The force between the liquid and the solid surface of the material, which causes the spreading of the liquid over the solid surface, can be either cohesive force or adhesive force, and it is expressed by the contact angle value (θ), allowing to identify the nature of the material surface [3,189]. The surface that attracts the water molecules is considered as hydrophilic and possesses high surface energy, whereas hydrophobic surfaces carry low-surface energy-repel water molecules. Several studies have confirmed that protein adsorptions are more likely to occur on hydrophobic surfaces, while cell adhesion and proliferation prefer hydrophilic surfaces [190,191,192]. The cell adhesion is reported to be enhanced on polymer surfaces with moderate wettability (θ = 40–70°) [193]. Through the changes created in surface chemistry and surface topography, the wettability of the surface can be adjusted from hydrophobic to hydrophilic or vice versa [194,195].

In a study, aligned polylactic acid (PLLA) nanofibrous scaffolds coated with graphene oxide after the aminolysis promoted the growth of Schwann cells (SCs), regulated cell orientation, and induced cell differentiation and neurite growth [196]. These scaffolds displayed good hydrophilicity and performance for nerve generation. A 3D printed functionally graded scaffold (FGS) made of PCL and β-TCP for the early stage treatment of osteonecrosis of the femoral head performed a surface treatment with sodium hydroxide (NaOH) (mercerization) to enhance the hydrophilicity and surface roughness of scaffolds [197]. Azido-modified polyether ether ketone (PEEK) biomaterial, biofunctionalised with antimicrobial peptide (AMP) and osteogenic growth peptide (OGP) via the bioorthogonal click reaction to obtain a dual-effect of host defence and tissue repair, revealed that the significant decrease in the water contact angle after the surface modification could be ascribed to the high hydrophilicity of (DOPA)6-PEG5-Azido and dibenzyl cyclooctyne (DBCO)-capped peptides [198]. Nanofibrous polyethersulfone (PESf) scaffolds fabricated by electrospinning were surface-modified by fluorapatite nanoparticles (FAn), showing higher hydrophilicity (complete wetting) than plasma-treated PESf, due to the highly hydrophilic nature of FAn decorated on the scaffold surface, improving stem cells behaviour and osteogenic activity in vitro [140]. PCL films surface coated with gelatin resulted in a lower contact angle, indicating improved hydrophilicity caused by the superficial bond formation regarding the surface modification role of gelatin; as a result, better cell adhesion, proliferation, and growth were achieved [199]. The plasma treatment technique has been widely explored for tailoring surface-wetting properties without altering the physicochemical features of the bulk material [200,201,202]. Air plasma treatment carried out on the PEEK and titanium surface exhibited an improvement in surface wettability [203]. A plasma treatment applied on the PCL/PEO blend electrospun nanofibres for the functionalisation of the surface with amino groups has influenced protein adhesion as well as hydrophilicity [204]. The hydrophobic surface created on the polyamide-6 nanofibrous scaffold after the decoration with hydroxyapatite nanoparticles (PA6/HAn scaffold) significantly improved the adsorption efficiency of vitamin D3, which is beneficial for bone growth and the prevention of osteoporotic fractures [205,206].

The UHMWPE surface functionalised with amino groups increased its wettability [159]. The hydrophilic surface provided by the -NH_2_ groups facilitated the adsorption of proteins from synovial fluid and thus improved boundary lubrication. The -NH_2_ groups incorporated into MBGs maintained the hydrophilic–hydrophobic balance, which is conducive to cell adhesion [181]. The glass surfaces functionalised with methyl, amino, and hydroxyl groups by silanation displayed that the hydrophobicity of the surface increased in the order of -OH << -NH_2_< -CH_3_ [207]. The hydrophobic surface modified with -NH_2_ and -CH_3_ suppressed the MDA-MB-231 cell adhesion and proliferation, inducing cell apoptosis, and mitochondria-mediated apoptosis by suppressing the phosphatase and TENsin homolog deleted on chromosome 10 (PTEN)/phosphoinositide 3-kinase (PI3K)/Ak strain transforming (AKT) pathway. Negatively charged CMCG composite membranes on titanium (Ti) substrates were produced via the AED-inhibited cell apoptosis of human BMSCs [183]. The presence of gelatin provided some degree of hydrophobic nature for the composite. PCL electrospun nanofibre scaffolds were modified with a highly hydrophilic PEG and a biocompatible block-co-polymer: poly(L-lactide-co-ε-caprolactone-co-glycolide) (PLCG), and resulted in the copolymers PCL-PLCG and PCL-PEG-PLCG scaffolds, which exhibited a super hydrophilic nature due to high porosity compared to PCL-PEG and PCL scaffolds [208].

It has been reported that the hydrophilic surface of implants encouraged early osseointegration by improving the early cellular response of bone-forming cells through increased adsorption of cell adhesion proteins [209]. Based on the extent of bone-to-implant contact (BIC), they found that the degree of osseointegration after four weeks was superior for the hydrophilic SLActive compared with the hydrophobic SLA surface. Among the surfaces modified with -CH_3_, -NH_2_, and -OH groups, a suitable wettability for osteogenesis on hDPSCs was offered by the surface of amino functionality, which possessed a moderate contact angle of ~56° [155]. Nanothin coatings, functionalised with four chemical groups by the plasma polymerization technique, were characterised to study the effect of surface wettability properties on human serum-derived protein corona formation on biomaterial surfaces [210]. The results showed that enhanced dysopsonin albumin on hydrophilic surfaces led to an increase in anti-inflammatory cytokine, while opsonin immunoglobulin (IgG2) adsorption observed on hydrophobic surfaces promoted proinflammatory cytokine production, respectively.

## 7. Biological Properties of Biomaterial

### 7.1. Functionalisation with Biomolecules

One effective method to increase the bioactivity of biomaterials and achieve optimal tissue integration is to functionalise them with cell instructive molecules from the ECM [211]. The surface functionalisation by mimicking the cellular microenvironment provides a reproduction of biochemical signals involved in the regeneration of tissue by incorporating biological cues that recapitulate the ECM of the target tissue [212]. Proteins, peptides, primarily the RGD cell adhesive motif, and growth factors have been widely employed to functionalise biomaterials for tissue regeneration because of their ability to control cell behaviour [213].

A bioactive antifouling vascular graft bearing a biofunctional peptide was developed using hierarchical polymer brushes, and it demonstrated specific ECs adhesion and proliferation, opening the possibility of endothelialize artificial conduits [214]. In this study, they created hierarchical diblock poly (methyl ether oligo (ethylene glycol) methacrylate-block-glycidyl methacrylate) brushes bearing azide groups (poly (MeOEGMA-block-GMA-N3)), which were grown by surface-initiated atom transfer radical polymerization (SI-ATRP) and functionalised with biomimetic RGD peptide sequences. The aforementioned structure was adapted to enable the surface modification of grafts made of woven polyethene terephthalate (PET) fibres. A biomimetic peptide integrating the RGD cell adhesive sequence and the osteogenic DWIVA motif derived from the wrist epitope of bone morphogenetic protein-2 (BMP-2) was deposited on a glass surface and synergistically improved C2C12 adhesion, inhibited myoblast differentiation, and activated p38 expression [215]. An increase in wettability for this surface was detected, which arises from the presence of charged and polar amino acids in the peptide sequence, capable of creating hydrogen bonds with the water droplets. Based on polycaprolactone-co-lactide (PCLLC) scaffolds conjugated with DOPA-containing peptide from blue mussel (MP), which were equipped with bioactive integrin peptides and PG binding sites (FHRRIKA), a multifunctional modular assembly was developed that served as a suitable biomimetic coating for the cardiovascular devices [216]. Under static and fluidic environments, the immobilisation of the bioactive peptides by catechol-mediated surface binding enhanced endothelial adhesion. The bifunctional peptide coating outperformed the unspecific adsorbed adhesion proteins like collagen I. In addition, integrin signalling promoted cell survival and differentiation, which were strengthened by C-X-C Motif Chemokine Ligand 12 (CXCL12) and vascular endothelial growth factor (VEGF) delivery. A nanoscale modification in which RGD nanopatterns were applied on a non-fouling background of PEG examined on human umbilical vein endothelial cells (HUVECs) displayed a better cell adhesion on the surfaces of RGD nano spacing less than 70 nm and exhibited a monotonic decrease of adhesion with the increase of RGD nano spacing, while cell migration on the nanopatterned substrates exhibited a nonmonotonic trend that peaked at 91 nm of nano spacing [217]. PEB scaffolds in which the bone marrow-derived MSC-specific affinity peptide E7 and a BMP-2 mimetic peptide were concomitantly conjugated onto PCL polymer revealed that the scaffold could synchronously promote adhesion and osteogenic differentiation of bone marrow-derived MSC as a result of the co-delivery of E7 and BMP-2 mimetic peptides [218]. Immobilisation of RGD on chitosan scaffolds, which were incorporated with PLGA-PEG and β-TCP nanoparticles, showed good hydrophilicity and biocompatibility, thus supporting cell adhesion and growth [219].

The fabrication of a scaffold for TE based on the self-assembling potential of a bioactive peptide, inspired by the native tenascin-C protein, has been explored recently [220]. This peptide sequence demonstrated a high propensity to form a nanofibrous network at physiological pH due to its ideal hydrophilic–lipophilic balance. This nanofibrous network then entangled to form a higher-ordered structure, leading to a supramolecular hydrogel formation, which mimics the natural nano-architecture of ECM. With the classic cell adhesion peptide motif CYGGGRGDSK(biotin) (RGDS(biotin)) and its negative control CYGGGRGESK(azide) (RGES(azide)) having already been modified with the biorthogonal groups like biotin, and azide, peptide-PCL conjugates were created and 3D printed into scaffolds with one or both peptides [221]. The outcomes showed that both the spatial control over peptide functionalisation and the peptide concentration on the surface of the 3D-printed fibre had an impact on the level of cell attachment. Scaffolds printed with the greatest RGDS (biotin)-PCL concentrations had a considerable increase in NIH3T3 fibroblast adhesion, and cells preferentially adhered and spread on RGDS (biotin)-PCL fibres over RGES (azide)-PCL fibres. A composite alginate/fluorenylmethoxycarbonyl-diphenylalanine (FmocFF) hydrogel as an injectable scaffold, fabricated for bone regeneration, exhibited a similarity towards GAGs/fibrous proteins, respectively, which are the main macromolecules composing the ECM, facilitating the adhesion, proliferation, and osteogenic differentiation of MCT3T-E1 preosteoblasts [222]. It has been reported that the PC12 cells cultured on an electrically stimulated p(Lys)long g pellet were demonstrated to have improved adhesion and neurite outgrowth. These conductive and biocompatible Pep g materials linked by covalent amide bonds can be used to direct stem cell differentiation [223]. LLP2A, a high-affinity peptidomimetic ligand was grafted onto the PLLA/PCL electrospun microfibrous scaffolds, confirming that LLP2A had a strong binding to human early gestation chorionic villi-derived MSCs (CV-MSCs) via integrin α4β1 and LLP2A modification significantly increased CV-MSC adhesion, spreading and viability on the polymeric scaffolds via regulating signalling pathways including phosphorylation of focal adhesion kinase (FAK) and AKT, nuclear factor kappa B (NF-kB) and Caspase 9 (CASP9) [224]. Laminin, a neurite-promoting protein, has been used to modify PLGA/carbon nanotube (CNT) electrospun nanofibrous scaffolds via either mussel-inspired poly(dopamine) (PD) coating or physical adsorption, revealing that PLGA/CNT-PD-Lam scaffolds preserved laminin for a longer time and promoted neurite outgrowth compared to PLGA/CNT-Lam and unmodified scaffolds [225,226].

### 7.2. Biocompatibility

The acceptance of biomaterials by the body is fundamentally related to their success, which can occur with the suitable surface of the biomaterial, which in turn usually makes first contact with cells and tissue [227]. Therefore, the biomaterial’s surface ought to prevent any unfavourable side effect in the recipient or beneficiary, such as damage, cytotoxicity, genotoxicity, mutagenicity, carcinogenicity, or immunogenicity, and should instead promote more vital cellular responses and envisioned functions related to the medical treatment [228].

In addition to having good biocompatibility for HUVEC cells, a copolymerized coating of dopamine and hexamethylenediamine (PDAM/HD), rich in amino groups and applied to a stainless-steel surface, attenuated tissue response with less inflammatory cell infiltration, granulation tissue formation, and thinner fibrous capsule development [229]. The thiol and amine group-functionalised MBG scaffolds showed good biocompatibility and also possessed good apatite mineralisation ability [162]. The hDPSCs attached to the amino-functionalised surface of the SAMs not only improved the osseointegration of dental implant materials but also exhibited good biocompatibility, proving applications in bone graft or plastic surgery [155]. PCL 3D printed scaffolds fabricated through surface aminolysis and layer-by-layer techniques accelerated the vascular pattern formation of human umbilical ECs and boosted the mineralised matrix production and the expression of osteogenesis-related genes during osteogenic differentiation of MSCs in in vitro studies [230]. CMCG composite membranes on Ti substrates stimulated cell proliferation and adhesion of BMSCs, and showed good biocompatibility for in vitro studies [183].

Electrospun silk/melanin nanofibrous scaffolds have supported the human neuroblastoma cell attachment and viability, thereby confirming their biocompatible nature, and offering an effective candidate for nerve regeneration and recovery [231]. Amine plasma-polymerization performed on the maxillofacial Ti plates used in clinical surgery positively influenced the osteoblast cell behaviour, such as proliferation, and differentiation, and proved to be more biocompatible because of the hydrophilic amino groups [232]. Polyhedral oligomeric silsesquioxane (POSS) nanoparticles were introduced into a PEG hydrogel to prepare a POSS–PEG hybrid hydrogel, and then coated on the surface of a decellularized heart valve (DHV) to prepare the composite scaffold, reporting good blood compatibility, excellent cell compatibility, and promoting cell adhesion and proliferation, suggesting an alternative scaffold material with anti-calcification potential for an artificial heart valve [233,234].

### 7.3. Biodegradability

It has been shown that the fundamental physical and chemical characteristics of polymeric materials play a significant role in how biodegradable they are [235]. The rate of biodegradability depends on the crystallinity and surface wettability of the biomaterial surface. The biodegradable polymers based on polyesters, such as Poly(D,L-lactic acid) (PDLLA), PCL, and Poly(glycolic acid) (PGA), seem to be promising candidates due to their good biocompatibility and, as a result, they have been gaining attention as environmentally friendly alternatives to be used in medicine [236].

Poly(3-hydroxybutyrate-*co*-3-hydroxyvalerate) (P(3HB-*co*-3HV)) was functionalised with ascorbic acid through lipase-mediated esterification. The obtained copolymer P(3HB-*co*-3HV)-ascorbic acid behaved as an antioxidant-active biomaterial and showed a 1.6-fold increase in its biodegradability as compared to the non-functionalised P(3HB-*co*-3HV) [237]. The hydrophilicity of the surface produced by functionalising ascorbic acid was credited with the higher biodegradability rate. Biologically active hydrophilic moieties like sugars have been explored for the functionalisation of synthetic polymers [238,239]. Polyhydroxyalkanoates (PHA), a well-known class of aliphatic biopolymers, were functionalised with sucrose by lipase-based catalysis, and the biodegradability of the resulting copolymer, poly(1’-O-3-hydroxyacyl-sucrose), was found to be around 1.5-times greater than that of the non-functionalised polymer [240]. Nanocomposites based on poly(3-hydroxybutyrate-*co*-3-hydroxyvalerate) (PHBV) were prepared by the incorporation of graphite nanosheets (GNS) using a solution casting method, which showed a complete degradation in the presence of *Penicillium funiculosum* [241].

## 8. Conclusions and Perspectives

TE and RM are gaining more and more interest in the treatment of degeneration or loss of organ and/or tissue function due to injury, disease, or ageing. These approaches are based on the design and manufacture of scaffolds, 3D biodegradable and biocompatible structures that mimic the characteristics of the native tissue and the nano-architecture of the native ECM, promoting new tissue regeneration. For this reason, it is necessary to understand composition, structure, and functions of the native ECM.

In this field, scaffolds can be fabricated and functionalised to better mimic the target tissue in which they would act. Nowadays, many studies provide a huge quantity of information about scaffolds’ modifications to improve cell–biomaterial interactions. Paramount in this field is the definition of UCPs, which can describe these interactions in a simple way and can provide a new methodology to understand how scaffolds interact with cells and therefore permit to immediately identify critical aspects that can be tailored in the production of scaffolds.

In general, all physical and chemical properties of both scaffolds’ bulk and surface could be essential to create a bond between cells and biomaterial and promote tissue regeneration. This review aimed to provide advances in the knowledge of cell–biomaterial interactions by discussing studies and new findings in this field. It is also necessary to underline the importance of keeping in mind the tissue of interest to select only those parameters that can effectively enhance the efficiency of the scaffold because the same modification could be effective in some cases and have negative effects in others.

In conclusion, it can be observed how a deep knowledge of what happens in the interaction between cells and biomaterials can lead to innovative and optimal strategies in TE and RM, which can be translated into clinical applications shortly. Although they represent a promising way, their use in human patients is still difficult due to the complex interactions between ECM and biomaterials and the lack of knowledge about them and the possible materials and techniques that can be used. For this reason, continuous research by the scientific community in this field is needed.

## Figures and Tables

**Table 1:** Modification techniques and their impact on cell–biomaterial interaction.

Modification Techniques	Materials	Cell Responses	References
Layer-by-layer assembly	HP and CS-coated PU/DCS scaffolds	Promoted cell attachment and proliferation of endothelial progenitor cells and long, in vitro coagulation time, and high resistance to platelet adhesion.	[90]
	BP-NS/CS composite-coated PEEK scaffolds	Enhanced biocompatibility and osteogenesis-associated gene expression.	[91]
	HP/Collagen encapsulating NGF coated on PLLA scaffolds	Promoted and directed SCs growth as well as induced the differentiation of PC12 cells and neurite growth along the nanofibrous alignment.	[92]
Nanoparticle assembly	Au NPs on PLGA nanofibrous sheet	Enhanced the osteogenic differentiation of human adipose-derived stem cells and biocompatibility.	[93]
	PDA NPs on TCP scaffolds	Demonstrated excellent osteoinductivity and bone-regeneration performance.	[94]
	SF NPs on PLLA Scaffolds	Excellent adhesion, proliferation, and osteogenic differentiation on MC3T3-E1 cells and induced a higher level of osteoblast-specific markers.	[95]
Electrospinning	Core-shell SF/PCL/PVA nanofibrous with CTGF and BMP2	Excellent improvement in vessel formation and bone tissue recovery and pro-angiogenic effect on bone healing.	[96]
	PCL/PDS scaffolds	Improved hydrophilicity, a significant increase in proliferation of HUVECs, faster cellularization, and better vascularization.	[97]
	PCL/GLA nanofibrous with WS NPs	Showed excellent viability, growth, and proliferation of ASCs.	[98]
UV treatment	GLA nanofibrous scaffolds	Promoted adhesion and proliferation of HaCaT, without causing apparent cytotoxicity and induced a rapid cell migration close to 79% of an artificial wound within 24 h.	[99]
	PVP-PGS blend fibres	Exhibited good viability and proliferation of human dermal fibroblast cells.	[100]
	PV-Ci nanofibers modified with laminin peptides	Enhanced neural adhesion, outgrowth, and regeneration.	[101]
Laser treatment	PLGA- Collagen hybrid constructs	Exhibited good adhesion, and proliferation on HCECs and HKs and maintained their respective phenotypes well. HCECs could form multilayers.	[102]
	nHA loaded core–shell PCL/PCL and PCL/PVAc nanofibrous scaffolds	Showed high viability, very low mortality, and improved human osteoblast adhesion and proliferation.	[103]
Plasma treatment	PCL nanofibres treated with argon plasma	Enhanced metabolic activity, adhesion, and proliferation of ADSCs.	[104]
	PLLA/Baghdadite scaffold treated with oxygen plasma	Induced osteogenesis-related genes and enhanced osteogenic differentiation of AD-MSCs.	[105]
	PCL/GLA nanofibres treated with cold atmosphere plasma	Improved cell affinity, growth adhesion, and proliferation of MSCs.	[106]
Cross-linked assisted adsorption	PCL/GAGs Scaffolds (EDC/NHS)	Improved adhesion, proliferation, and differentiation of SCs.	[107]
	Keratin/PEO/nHa nanofibrous membrane (EGDE)	Enhanced the proliferation of L929 cells, hence exhibited an advantage in reducing the inflammatory response in the infective stage and enhancing skin repairing processes in the following recovery stages.	[108]
	PCL/GLA/FG scaffolds (GA)	Enhanced hCB-ECs growth and improved maintenance of their EC phenotype in vitro.	[109]
Wet chemical techniques	PCL nanofibres (Hydrolysis-NaOH)	Improved protein adsorption and attachment, viability, and elongation of 3T3 fibroblasts.	[110]
	PCL/Maltose nanofibres	Showed higher proliferation and better morphology of the HUF cells.	[111]
	PAN/Fibrin (Hydrolysis-NaOH)	Increased adhesion and proliferation of HUVECs and promoted endothelialisation.	[112]
Molecular imprinting	GLA/nHA scaffolds	Promoted osteogenesis of hMSCs and induced the formation of a stable vascular network in the HUVEC-laden hydrogel.	[113]
	Peptide imprinted Alg/GLA/Ela sponges	Improved cardiac progenitor cell adhesion and differentiation toward myocardial phenotypes.	[114]
	Tenocyte imprinted PDMS	Induced significant tenogenic differentiation on ADSCs.	[115]
Click chemistry	CM-2 immobilised HA hydrogel	Enhanced chondrogenic differentiation of hPLSCs.	[116]
	HEC/CA scaffolds	Improved biocompatibility, chondrogenic ability, and potential for cartilage repair and regeneration.	[117]
	Gellan hydrogels	Promoted MSCs adhesion and metabolic activity.	[118]

Abbreviations: AD-MSCs: adipose tissue-derived mesenchymal stem cells, ADSCs: adipose-derived stem cells, Alg: Alginin, Au NPs: Gold nanoparticles, BMP2: bone morphogenetic protein 2, BP/NS: Black phosphorous nanosheets, CA: Citric acid, CM: Cytomodulin, CS: Chitosan, CTGF: connective tissue growth factor, DCS: Decellularized scaffold, EDC: 1-ethyl-3-(3 dimethylaminopropyl) carbodiimide hydrochloride, EGDE: ethylene glycol diglycidyl ether, Ela:Elastin, FB: Fibrinogen, GA: Glutaraldehyde, GAG: Glycosaminoglycan, GLA: Gelatin, HA: hyaluronic acid, HaCaT: human keratinocytes, hCB-ECs: human cord blood-derived endothelial cells, HCECs: human corneal epithelial cells, HEC: Hydroxy ethyl cellulose, HKs: human keratocytes, hMSCs: human mesenchymal stem cells, HP: Heparin, hPLSCs: human periodontal ligament stem cells, HUFs: human uterine fibroblast cells, HUVECs: human umbilical vein endothelial cells, MSCs: mesenchymal stem cells, NaOH: Sodium hydroxide, NGF: nerve growth factors, nHA: nanohydroxyapatite, NHS: *N*-hydroxysuccinimide, TCP: β-Tricalcium phosphate, PAN: Polyacrylonitrile, PCL: polycaprolactone, PDA/NPs: polydopamine nanoparticles, PDS: polydioxanone, PDMS: Polydimethylsiloxane, PEO: polyethylene oxide, PEEK: Polyetheretherketone, PGS: Poly (glycerol sebacate) PLGA: Poly (lactide-co-glycolic acid), PLLA: poly (L-lactic acid), PU: polyurethane, PVA: poly(vinyl alcohol), PVAc: polyvinylacetate, PV Ci: polyvinyl cinnamate, PVP: Polyvinylpyrrolidone, SCs: Schwann cells, SF NPs: silk fibrin nanoparticles, WS NPs: lignocellulosic nanoparticles from walnut shells.

## Data Availability

Not applicable.
